# Intercalation of Ionic Liquids into LDH Structures for Microwave-Accelerated Polymerizations

**DOI:** 10.1021/acs.inorgchem.3c02021

**Published:** 2023-08-28

**Authors:** Hynek Beneš, Magdalena Konefał, Sonia Bujok, Ondřej Mrózek, Ewa Pavlova, Darina Smržová, Petra Ecorchard

**Affiliations:** † Institute of Macromolecular Chemistry of the Czech Academy of Sciences, Heyrovského nám. 2, 16200 Prague 6, Czech Republic; ‡ Institute of Inorganic Chemistry of the Czech Academy of Sciences, Husinec-Řež 1001, 25068 Řež, Czech Republic

## Abstract

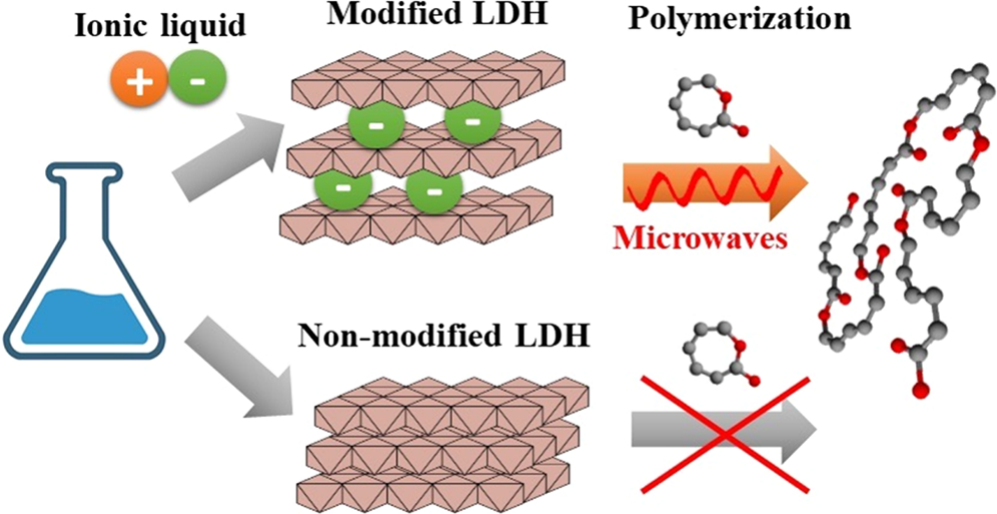

Microwave-accelerated ring-opening polymerization (ROP) of cyclic esters catalyzed by ionic liquid (IL) anions, intercalated into layered double hydroxides (LDHs), has been recently described as a fast and environmentally friendly synthetic way to prepare biodegradable polyester/LDH nanocomposites. However, to observe this synergistic catalytic effect between microwaves and IL anions and to achieve a homogeneous structure of the final polymer nanocomposite, the IL anions must be efficiently intercalated inside the LDH structure. Herein, we investigate the effects of various metal compositions of M^2+^/Al^3+^ LDHs (M = Mg, Co, and Ca) and different LDH synthetic routes (one-step direct coprecipitation, two-step coprecipitation/anion exchange, and two-step urea/anion exchange) on the intercalation efficiency of trihexyltetradecylphosphonium bis(2,4,4-trimethylpentyl)phosphinate IL. The most effective IL anion intercalation was observed for Ca^2+^/Al^3+^ LDH prepared using the two-step method consisting of coprecipitation and subsequent anion exchange. After optimization, this synthetic pathway led to the production of LDHs with intercalated IL anions and a reduced amount of intercalated water (<0.6 wt %). The catalytic ability of thus optimized LDH particles was demonstrated on the microwave-assisted ROP of ε-caprolactone, showing rapid progress of polymerization. Within minutes, the polycaprolactones with an average molecular mass in the range of 20 000–50 000 g/mol containing fully delaminated and exfoliated LDH nanoparticles were obtained.

## Abstract 2

Modified M^2+^/Al^3+^ LDHs with intercalated phosphate anions from a phosphonium ionic liquid were prepared. The effectiveness of intercalation was studied for various M^2+^ compositions (M = Mg, Co, and Ca) of LDHs using three different synthetic routes (one-step direct coprecipitation, two-step coprecipitation/anion exchange, and two-step urea/anion exchange). The modified LDHs exhibited a catalytic effect during microwave-accelerated ring-opening polymerization of ε-caprolactone.

## Introduction

Layered double hydroxides (LDHs), also known as hydrotalcite-like compounds or anionic nanoclays, have two-dimensional structures represented by the following general formula^1^
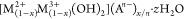
where M^2+^ and M^3+^ are divalent (Mg^2+^, Cr^2+^, Ca^2+^, Fe^2+^, Mn^2+^ or Co^2+^) and trivalent (Al^3+^, Co^3+^, Fe^3+^, Cr^3+^ or Mn^3+^) cations, respectively, A^*n*–^ is an anion (carbonate, nitrate, chloride or bromide), and *x* is the molar ratio of M^3+^/(M^2+^ + M^3+^), typically in the range of 0.2–0.33.^2^ The M^2+^ and M^3+^ cations are linked by hydroxyl groups coordinated at the octahedral positions forming positively charged layers/sheets, which are balanced by exchangeable A^*n*–^ anions and water present in the interlayer region.^3^

Due to the highly variable and adjustable chemical structure and morphology, LDH particles have mainly been used as catalysts^4^ or catalyst supports,^5,6^ adsorbents,^7,8^ anion exchange materials, and fillers in nanocomposites,^9^ improving their fire-retardant properties,^10^ crystallization behavior,^11^ etc. The high compositional flexibility of LDHs, which can be tailored using different metal cations and interlayer compensating anions, results in a large variety of host–guest assemblies and nanoarchitectures with versatile physical and chemical properties.^4^ The unique ability to regenerate a layered structure after calcination enables the design and fabrication of novel composite materials with intercalated functional guest anions. In particular, immobilized active species demonstrate enhanced catalytic activity, selectivity, stability, and recyclability compared to their homogeneous analogues.^12^

Recently, ionic liquids (ILs) have been investigated as polymerization catalysts/initiators of ε-caprolactone (εCL),^9^ ε-caprolactam,^13^ and epoxy resins.^14−16^ ILs are organic salts whose melting point is below 100 °C; indeed, many are liquids at room temperature.^17,18^ IL molecules usually consist of a cationic “head” with an anionic counterion and one or more hydrophobic (aliphatic and/or aromatic) “tails.” Depending on the type of cationic core, the size and type of the counterion, the length of aliphatic alkyl chain(s), or a combination of alkyl chains with aromatic structures, ILs exhibit several intrinsic properties such as low volatility, high thermal and chemical stability, insignificant flammability, good thermal conductivity, high ionic mobility, stability in the presence of moisture, etc.^19−21^ Mainly thanks to beneficially high thermal stability and tuneable chemical structures, ILs have a huge potential for applications in the field of polymers, e.g., as initiators of polymerization,^22^ catalysts,^16^ curing agents,^23,24^ or building blocks of polymer networks.^25,26^ However, the high viscosity of ILs and their difficult separation from the products are the main limitations of their wider use in polymer catalysis.^27^

To overcome these drawbacks, the concept of heterogenization of ILs has been recently established, which combines the advantages of ILs (mainly high chemical and structural tunability) and LDH support (chemical and thermal resistance, encapsulation ability, etc.).^28^ Our previous study showed that ILs immobilized on LDH support significantly accelerated ring-opening polymerization (ROP) of ε-caprolactone (εCL).^9^ Interestingly, it was found that the IL-intercalated LDHs exfoliated completely during in situ microwave-assisted ROP owing to the synergistic effect of IL anions intercalated between LDH galleries and microwave irradiation.^29^ Buffet et al. demonstrated that the chemical composition of LDH support might dramatically affect catalyst activity, polymer morphology, and polymer microstructure.^30^ However, to date, there have been no reports about the effect of synthetic conditions for the preparation of the LDH structure on the immobilization of ILs.

LDHs can be synthesized using various methods including coprecipitation, urea hydrolysis, sol–gel process, hydrothermal synthesis, reformation, and mechanical milling, among others.^31^ The synthetic conditions (e.g., pH, aging time, temperature, and M(II)/M(III) ratio) strongly affect the morphology and physicochemical properties of the resulting LDH particles,^32^ which in consequence influence their key ability to intercalate a variety of molecules into the LDH galleries via anion exchange.^33^ It was demonstrated that a higher M(II)/M(III) ratio decreases the anion exchange. The thickness of the cation layer and the distance of the anion interlayer also directly affect the anion replacement. The coprecipitation method is widely used for LDH synthesis, which can be adopted as a one-step method for the direct synthesis of LDHs with intercalated IL anions. IL is mixed with the precursor salt of metal cations and is present during the course of precipitation. Difficult homogenization of the reaction mixture leading to concentration inhomogeneities of the precipitating agent, formation of aggregates, and poor crystallinity of the formed LDHs are the main drawbacks of this method.^34,35^ The urea method produces LDH sheets with large lateral dimensions and high crystallinity. The decomposition of urea into carbonate anions having a high affinity toward positive LDH layers makes this method challenging for the synthesis of IL anion-modified LDHs. Schwieger et al.^36^ were able to prepare LDHs with intercalated nitrate anions. The subsequent exchange of nitrate to other anions was then easier because of the lower affinity of nitrate anions toward LDH layers.

Herein, we investigate three synthetic routes (one-step direct coprecipitation, two-step coprecipitation/anion exchange, and two-step urea/anion exchange), which lead to LDH particles with immobilized trihexyltetradecylphosphonium bis(2,4,4-trimethylpentyl)phosphinate as a representative phosphonium IL. The incorporation of IL into the LDH structure (surface modification vs intercalation) is evaluated concerning the synthetic procedure, type of M^2+^ cations (Mg, Co, Ca) in M^2+^/Al^3+^ LDHs, and the final structure of IL-modified LDHs. The effects of different M^2+^/Al^3+^ LDH compositions and various synthetic conditions on the intercalation efficiency of the phosphonium IL are evaluated, aiming to find an optimal LDH composition and synthetic conditions for the preparation of IL-intercalated LDH particles. Finally, the LDH particles with the highest degree of IL intercalation were tested as catalysts for microwave-accelerated ROP of εCL.

## Experimental Section

### Materials

Magnesium chloride hexahydrate, calcium nitrate tetrahydrate, aluminum nitrate nonahydrate, aluminum chloride hexahydrate, cobalt (II) chloride hexahydrate, methanol, nitric acid 65%, and hydrochloric acid 35% were supplied by Lach-Ner (Czech Republic). Sodium hydroxide, isopropyl alcohol (*i*PrOH), ethanol, urea, tetraethylammonium hydroxide (Et_4_NOH), and magnesium nitrate hexahydrate were provided by PENTA (Czech Republic) and Sigma-Aldrich (Czech Republic). Trihexyltetradecylphosphonium bis(2,4,4-trimethylpentyl)phosphinate (IL-P) was provided by IoLiTec (Germany). All chemicals were used without further purification.

### One-Step Coprecipitation (Direct) Synthesis

#### Synthesis of Mg^2+^/Al^3+^ LDH Modified with IL-P (D-MgAl-P)

A water solution of Mg(NO_3_)_2_·6H_2_O (1.282 g, 5 mmol), Al(NO_3_)_3_·9H_2_O (0.938 g, 2.5 mmol), and IL-P (1.00 g) in 100 mL of degassed *i*PrOH was preheated to 80 °C, and 20 mL of 20 wt % Et_4_NOH (precipitation agent exhibiting excellent solubility in alcohols and a suitable source of OH^–^ nucleophiles) in methanol was added dropwise via a cannula under an inert atmosphere. The mixture was refluxed overnight. After that, the solvent was filtered off, and the crude product was washed with ethanol and dried to receive a white powder of modified Mg^2+^/Al^3+^ LDH (12 h, 80 °C).

#### Synthesis of Co^2+^/Al^3+^ LDH Modified with IL-P (D-CoAl-P)

An ethanol–water (150 mL, 1:3 v/v) solution of NaOH (2.75 g) and IL-P (5.00 g) was titrated with a water solution of CoCl_2_·6H_2_O and AlCl_3_·6H_2_O at a Co/Al ratio of 2 (total metal ion concentration = 0.375 M) at 80 °C under an inert atmosphere. The obtained precipitate was filtered under reduced pressure, washed with ethanol, and dried to receive a blue powder of modified Co^2+^/Al^3+^ LDH (12 h, 80 °C).

#### Synthesis of Ca^2+^/Al^3+^ LDH Modified with IL-P (D-CaAl-P)

The water solution of Ca(NO_3_)_2_·4H_2_O and Al(NO_3_)_3_·9H_2_O at a Ca/Al ratio of 2 (total metal ion concentration = 0.375 M) was added dropwise to 150 mL of a preheated (80 °C) ethanol–water (1:3 v/v) solution of NaOH (2.75 g) and IL-P (5.00 g) placed in a flask equipped with a condenser and a nitrogen inlet. The obtained dispersion was aged for 1 h at the reaction temperature. Then, the sludge was filtered, washed with ethanol, and dried to receive a white powder of modified Ca^2+^/Al^3+^ LDH (12 h, 80 °C).

### Two-Step Coprecipitation/Anion Exchange Synthesis

#### Synthesis of Mg^2+^/Al^3+^ LDH Modified with IL-P (C-MgAl-P)

##### 1st Step of Coprecipitation

The water solution (150 mL) of NaOH (69 mmol) and HCl (34 mmol) was slowly titrated with an ethanol–water (150 mL, 1:3 v/v) solution of MgCl_2_·6H_2_O and AlCl_3_·9H_2_O (Mg/Al = 2, cation concentration = 0.375 M) at 80 °C under an inert atmosphere. Then, the obtained dispersion was aged at the same reaction conditions for 1 h. The sludge was filtrated on a Büchner funnel, washed with water, and dried at 80 °C for 12 h to receive a white powder of non-modified Mg^2+^/Al^3+^ LDH (C-MgAl).

##### 2nd Step of Anion Exchange

C-MgAl was first calcinated at 500 °C for 24 h. Thus, calcinated C-MgAl dispersed in water (1 g of LDH/100 mL of water) was titrated with an ethanolic solution of IL-P in an amount 1.5 times higher concerning the anion exchange capacity (3.35 mequiv/g, 3.83 g). The mixture was kept under nitrogen with constant stirring for 24 h at 60 °C. Then, the obtained sludge was filtered, washed with ethanol three times, and dried (12 h, 80 °C) to receive a white powder of C-MgAl-P.

#### Synthesis of Co^2+^/Al^3+^ LDH Modified with IL-P (C-CoAl-P)

##### 1st Step of Coprecipitation

The water solution of NaOH (69 mmol) and HCl (34 mmol) was titrated with an ethanol–water solution of CoCl_2_·6H_2_O and AlCl_3_·6H_2_O (cation concentration = 0.375 M, Co/Al = 2) at 80 °C under a nitrogen atmosphere. The dispersion was aged for 1 h at constant temperature (80 °C), filtered, washed with water, and dried at 80 °C for 12 h to receive a blue powder of non-modified Co^2+^/Al^3+^ LDH (C-CoAl).

##### 2nd Step of Anion Exchange

It was conducted similarly to the modification of Ca^2+^/Al^3+^ LDH. Water dispersion of non-calcinated C-CoAl was titrated with the ethanol solution of IL-P (100% excess of AEC, 3.35 mequiv/g, 5.1 g). After the aging period (24 h, 60 °C), the sludge was separated on a Büchner funnel, washed with ethanol, and dried (12 h, 80 °C) to receive a blue powder of C-CoAl-P.

#### Synthesis of Ca^2+^/Al^3+^ LDH Modified with IL-P (C-CaAl-P)

##### 1st Step of Coprecipitation

The ethanol–water (1:3 v/v) solution of Ca(NO_3_)_2_·4H_2_O and of Al(NO_3_)_3_·9H_2_O at a Ca/Al ratio of 2 and a total metal ion concentration of 0.375 M was added dropwise to 150 mL of a water solution of NaOH (69 mmol) and HNO_3_ (34 mmol). A flask equipped with a condenser and a nitrogen inlet was placed in a preheated oil bath (80 °C). Dispersion of LDH was aged for 1 h at the reaction temperature. Then, the precipitate was filtered under reduced pressure, washed with water three times, and dried (12 h, 80 °C) to receive a white powder of non-modified Ca^2+^/Al^3+^ LDH (C-CaAl).

##### 2nd Step of Anion Exchange

Water dispersion of non-calcinated C-CaAl (1 g LDH per 100 mL of water) was titrated with an ethanolic solution of IL-P at 60 °C under an inert atmosphere. The amount of IL-P was calculated regarding AEC (3.35 mequiv/g, 3.83 g) with 50% excess. After 24 h, the dispersion was filtrated on a Büchner funnel, and the solid product was washed with ethanol three times and dried at 80 °C for 12 h to receive a white powder of C-CaAl-P.

### Two-Step Urea/Anion Exchange Synthesis (Urea Method)

#### Synthesis of Mg^2+^/Al^3+^ LDH Modified with IL-P (U-MgAl-P)

##### 1st Step of the Urea Method

A solution of Mg(NO_3_)_2_·6H_2_O (2.564 g, 10 mmol), Al(NO_3_)_3_·9H_2_O (1.876 g, 5 mmol), and urea (2.100 g, 35 mmol) in 1 L of water was heated at reflux under a Dimroth condenser for 24 h. After that, the white precipitate was centrifuged at 10 000 rpm for 5 min, washed with 1 L of water and then 100 mL of ethanol, and dried (12 h, 80 °C) to receive a white powder of non-modified Mg^2+^/Al^3+^ LDH (U-MgAl).

##### 2nd Step of Anion Exchange

U-MgAl was first calcinated at 500 °C for 24 h. Then, a solution of 500 mg of IL-P in 20 mL of degassed ethanol was added dropwise via a cannula into the water dispersion of the calcinated MgAl (500 mg of LDH per 10 mL of degassed water). The mixture was heated to reflux under a Dimroth condenser for 24 h. After that, the solvents were filtered off, and the product was washed with ethanol and dried (12 h, 90 °C) to receive a white powder of U-MgAl-P.

#### Synthesis of Co^2+^/Al^3+^ LDH Modified with IL-P (U-CoAl-P)

##### 1st Step of the Urea Method

A solution of CoCl_2_·6H_2_O (2.379 g, 10 mmol), Al(NO_3_)_3_·9H_2_O (1.876 g, 5 mmol), and urea (2.100 g, 35 mmol) in 1 L of water was heated at 97 °C under a Dimroth condenser for 48 h. After that, a white precipitate was filtered off, washed with 1 L of water and 100 mL of ethanol, and dried (12 h, 80 °C) to receive a blue powder of non-modified Co^2+^/Al^3+^ LDH (U-CoAl).

##### 2nd Step of Anion Exchange

A solution of 500 mg of IL-P in 20 mL of degassed ethanol was added dropwise via a cannula into the water dispersion of the non-calcinated U-CoAl (500 mg of LDH in 10 mL of degassed water). The mixture was heated to reflux under a Dimroth condenser for 24 h. After that, the solvents were filtered off, and the crude product was washed with ethanol and dried (12 h, 90 °C) to receive a white powder of U-CoAl-P.

The overview of the synthetic methods used to fabricate Mg^2+^/Al^3+^, Ca^2+^/Al^3+^, and Co^2+^/Al^3+^ LDH modified with IL-P is given in Table 1.

**Table 1:** Overview of Sample Preparation, Anchoring of IL-P, and Labeling of the Samples

LDH type	pristine LDH name	anions of starting salts	sample name modified by IL-P
One-step Direct Coprecipitation Synthesis			
Mg^2+^/Al^3+^		NO_3_^–^	D-MgAl-P
Co^2+^/Al^3+^		Cl^–^	D-CoAl-P
Ca^2+^/Al^3+^		NO_3_^–^	D-CaAl-P
Two-step Coprecipitation/Anion-Exchange Synthesis			
Mg^2+^/Al^3+^	C-MgAl	Cl^–^	C-MgAl-P
Co^2+^/Al^3+^	C-CoAl	Cl^–^	C-CoAl-P
Ca^2+^/Al^3+^	C-CaAl	NO_3_^–^	C-CaAl-P
Two-step Urea/Anion-Exchange Synthesis			
Mg^2+^/Al^3+^	U-MgAl	NO_3_^–^	U-MgAl-P
Co^2+^/Al^3+^	U-CoAl	Cl^–^/NO_3_^–^	U-CoAl-P
Ca^2+^/Al^3+^	a	-	-

a The synthesis of U-CaAl LDH was not successful.

### Ring-Opening Polymerization of ε-Caprolactone in a Microwave Reactor

Microwave-assisted ROP of εCL was carried out in a monomodal microwave reactor Discover SP Microwave synthesizer (CEM Corporation) operating at 2450 MHz frequency. First, a mixture of εCL and LDH was placed (under an argon atmosphere) into a hermetically sealed (PTFE-silicon cups) 10 mL flask and treated with bath ultrasound for 10 min. Then, the flask was put into the microwave reactor and heated using a constant power of 30 W. During the progress of microwave heating, the temperature was continuously monitored using a built-in infrared thermometer.

### Characterization

Attenuated total reflection Fourier transform infrared (ATR-FTIR) spectra were recorded on a Spectrum 100T FTIR spectrometer (PerkinElmer) with a DTSG detector fitted with a Universal ATR accessory with a diamond/ZnSe crystal. All spectra were recorded in the range of 650–4000 cm^–1^ at 16 scans per spectrum and 4 cm^–1^ resolution. X-ray diffraction (XRD) patterns of the samples were collected with a diffractometer Bruker D2 equipped with a conventional X-ray tube (Cu Kα radiation, 30 kV, 10 mA). The primary divergence slit module width was 0.6 mm, Soller Module 2.5, air scatter screen module 2 mm, Ni Kβ-filter 0.5 mm, step 0.00405°, and time per step 0.3 s with a LYNXEYE 1-dimensional detector were used. Small-angle X-ray scattering (SAXS) measurements were performed using a Rigaku pinhole camera (modified molecular metrology system) attached to a Rigaku MicroMax 003 micro-focused X-ray beam generator, operating at 50 kV and 0.6 mA. The camera was equipped with a vacuum-compatible version of the Pilatus3 R 300 K hybrid photon-counting detector. A sample-to-detector distance of 1540 mm and an exposure time of 3600 s were used. The scattering vector *q* is defined as *q* = 4π/λ·sinΘ (λ – wavelength, 2Θ – scattering angle). The experimental setup covered a *q* range of 0.005–0.18 Å^–1^. The experiments were conducted at ambient temperature. Thermogravimetric analyses (TGA) of the samples were performed using a PerkinElmer Pyris 1 TGA in the temperature ranges of 30–650 °C (Mg^2+^/Al^3+^ LDH) and 30–750 °C (Ca^2+^/Al^3+^ LDH) at a rate of 10 °C/min; the purge gas flow rate was fixed at 25 mL/min nitrogen. The standard deviation of TGA measurement was under 5%. The high-resolution scanning electron microscopy (HRSEM) images were collected on an FEI Nova NanoSEM 450 scanning electron microscope in a high resolution with 10 kV acceleration voltage; a water suspension of samples was dropped on a Si wafer chip. Transmission electron microscopy (TEM) microphotographs were performed on a Tecnai G2 Spirit Twin 12 microscope (FEI, Czech Republic) in the bright field mode at the acceleration voltage of 120 kV. Ethanolic dispersions of nanoparticles prepared in an ultrasonic bath (3 min) were dropped on the microscopic Cu grids and subsequently covered with electron-transparent carbon film. The polymer yield was determined gravimetrically after extraction of the polycaprolactone (PCL) product with distilled water (three extraction cycles for 20 min at room temperature). Size exclusion chromatography was used for the determination of the number average (*M*_n_) and weight average (*M*_w_) molar mass as well as dispersity (*M*_w_/*M*_n_) of PCL. A GPC system equipped with a refractive index detector (Shodex, Japan) and a set of three columns (PLgel with a particle size of 10 μm, pore size: 50/10 × 103/10 × 104 Å, 300 × 7.5 mm^2^, Polymer Laboratories, U.K.) was used. THF (1 mL min^–1^) was used as a mobile phase. Calibration was done on the PS standards.

## Results and Discussion

### Synthesis of MgAl LDH Modified with IL-P

**Figure 1:**
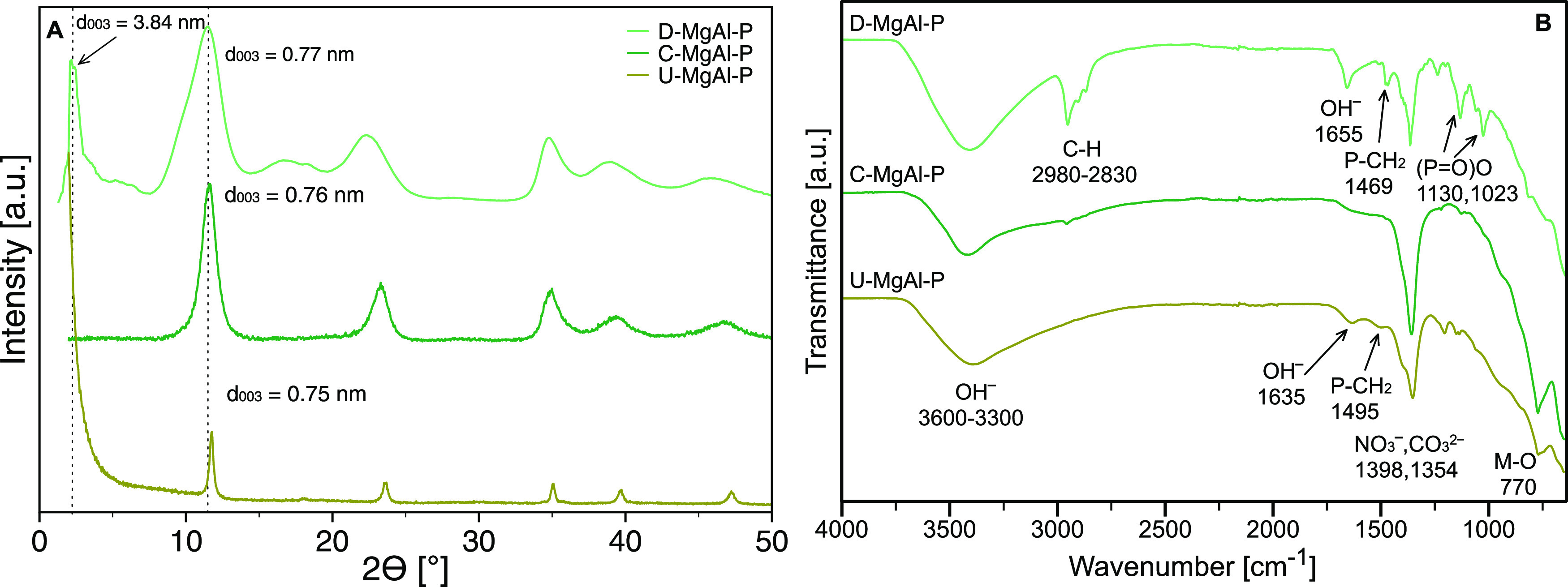
XRD pattern (A) and FTIR spectra (B) of MgAl LDH modified with IL-P (D—one-step direct coprecipitation, C—two-step coprecipitation/anion exchange, and U—two-step urea/anion exchange method).

The XRD patterns of the MgAl-P synthesized by three different methods (D—one-step direct coprecipitation, C—two-step coprecipitation/anion exchange, and U—two-step urea/anion exchange method) are compared in Figure 1A. The diffraction lines ( 2θ of ∼11.7, ∼23.6, and ∼34.8°), which match well with Mg^2+^/Al^3+^ LDH diffraction lines (003), (006), and (012),^37,38^ were displayed as relatively broad peaks, respectively.

The largest broadening was seen for D-MgAl-P, where the XRD pattern additionally contains an intense 2θ peak at 2.3° (3.84 nm), proving the intercalation of IL-P ions inside LDH galleries. However, the presence of an additional peak at 11.5°, corresponding to the (003) plane of the LDH phase with CO_3_^2–^ anions, and its higher-ordered reflections (006, 009, 0012) were the evidence of only partial intercalation. This was further supported by the peak broadening and asymmetry visible in the XRD pattern in the cases of the LDH phase intercalated with IL-P or with CO_3_^2–^ anions, which could be explained by corresponding reflections being close to each other and potentially overlapping. Although the direct synthesis was carried out in an inert atmosphere using nitrates as the starting salts, the produced LDHs were partially intercalated with CO_3_^2–^ anions as the result of their higher affinity toward the positive LDH layers than NO_3_^–^ anions.^39^

The pristine LDH prepared via the urea method (U-MgAl) was also prepared from NO_3_^–^ salt. However, the nitrate anion was also in this case replaced by the CO_3_^2–^ anion as evidenced by Figures 1A and S1A. The CO_3_^2–^ anion was present even after modification by IL-P for U-MgAl-P (*d*_003_ = 0.75 nm).

The Cl^–^ salts were used for the two-step synthesis of LDH via coprecipitation and subsequent anion exchange (C-MgAl-P). One can expect the slightly increased interlayer spacing in the XRD pattern thanks to the increasing ionic radius of Cl^–^ (ionic radius CO_3_^2–^ < NO_3_^–^ < Cl^–^),^40^ but the interlayer spacing of C-MgAl-P (*d*_003_ = 0.76 nm; 2θ = 11.6°) was nearly unchanged (Figures 1A and S2A), compared to D-MgAl-P (*d*_003_ = 0.77 nm; 2θ = 11.5°). Again, the intercalated CO_3_^2–^ anions were mainly present instead of the expected Cl^–^ anions in C-MgAl-P, even though the starting C-MgAl was calcinated and then modified under an inert atmosphere. The XRD trace of U-MgAl-P showed a potential peak at the very beginning of the diffractogram pattern. This peak was confirmed by the SAXS measurement (Figure S3), showing a broad reflection at 2θ = 1.2° corresponding to a d-spacing of 7.36 nm, which confirmed the intercalation of IL-P into the LDH structure.

FTIR spectroscopy was further used for verification of the presence of functional groups typical for IL-P.^41,42^ The FTIR spectrum of D-MgAl-P showed (Figure 1B) the intensive bands of C–H vibration at the range of 2830–2980 cm^–1^ and remaining less intensive vibration bands related to IL-P at 1469 cm^–1^ for P–CH_2_ (also for U-MgAl-P) and asymmetric and symmetric (P=O)O stretching vibrations at 1130 and 1023 cm^–1^, respectively (Figures 1B, S1B, and S2B),^41,42^ evidencing the presence of IL-P. The FTIR spectrum of C-MgAl-P (Figure 1B) showed very low intense bands of C–H vibration, which further confirms a very low efficiency of IL-P modification in this case.

### Synthesis of CoAl LDH Modified with IL-P

**Figure 2:**
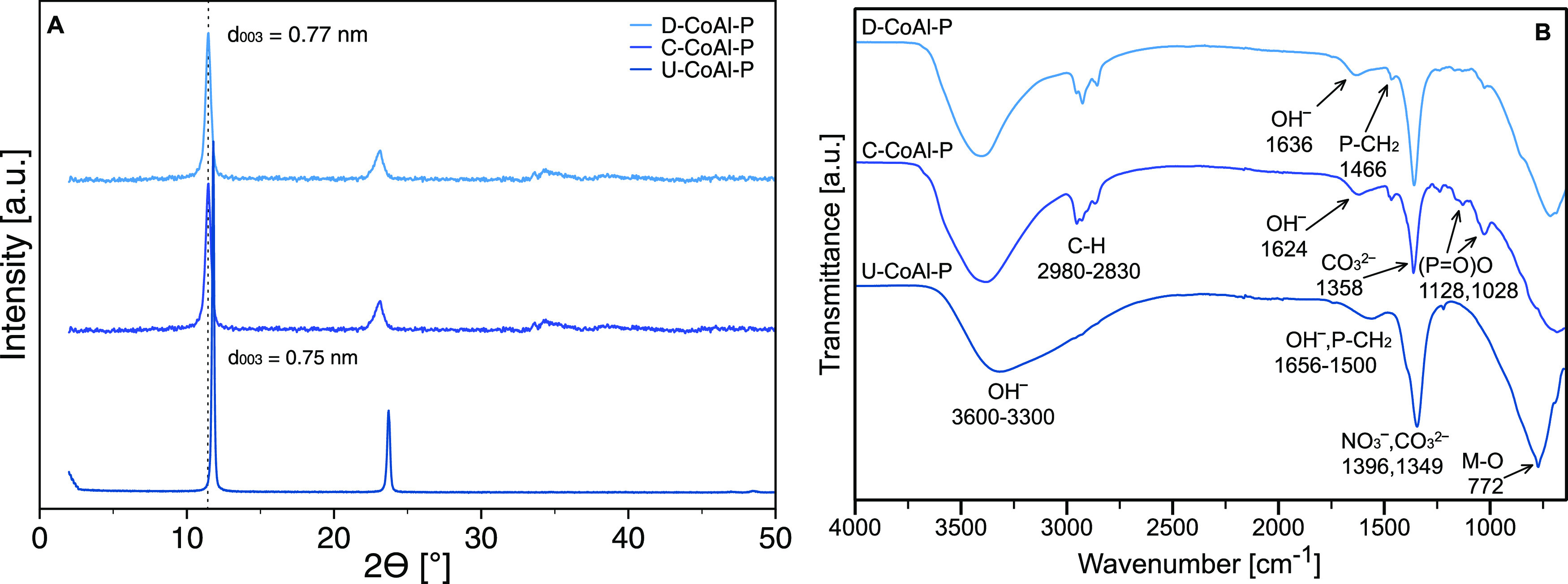
XRD pattern (A) and FTIR spectra (B) of CoAl LDH modified with IL-P (D—one-step direct coprecipitation, C—two-step coprecipitation/anion exchange, and U—two-step urea/anion exchange method).

In the case of IL-P-modified Co^2+^/Al^3+^ LDH, all three synthetic routes led to the production of highly crystalline products having an interlayer distance comparable with a basal spacing of the non-modified Co^2+^/Al^3+^ LDH (Figures 2A, S4A, and S5A). This indicated that the intercalation of organic anions of IL-P did not proceed. Possible reflection starting at a 2θ of∼2.6° in the XRD pattern of U-CoAl-P was refuted by SAXS measurements (Figure S3). The pristine U-CoAl was the only one prepared from the mixture of salts with Cl^–^ and NO_3_^–^ anions, while the others (D-CoAl-P and C-CoAl) were synthesized from the salts with Cl^–^ anions. No effect of the anion type of the starting salt on the presence of CO_3_^2–^ anions carbonate was observed. The (003) plane position (0.77 nm for D-CoAl-P and C-CoAl-P; 0.75 nm for U-CoAl-P) indicated that the LDH was exclusively intercalated by CO_3_^2–^ anions.^40^

The FTIR spectra of D-CoAl-P and C-CoAl-P (Figures 2B and S4B) showed vibration bands corresponding to IL-P as in the previous case of modified Mg^2+^/Al^3+^ LDH: the asymmetric and symmetric CH_2_ and CH_3_ vibrations at 2980–2830 cm^–1^, methylene deformation vibration P–CH_2_ at 1466 cm^–1^, and asymmetric and symmetric (P=O)O stretching vibrations at 1128 and 1028 cm^–1^. The relative intensities of all characteristic IL-P bands for both samples (D-CoAl-P and C-CoAl-P) were lower than those for D-MgAl-P but higher than those for C-MgAl-P and U-MgAl-P. The U-CoAl-P spectrum contained just the most intensive band for P–CH_2_ with low relative intensity. The band related to CO_3_^2–^ anions at 1358 cm^–1^ was also found in the FTIR spectra of D-CoAl-P and C-CoAl-P, thus affirming the exchange of chloride anions with carbonate anions, which were generated from atmospheric carbon dioxide dissolved in the reaction medium. The FTIR spectrum of U-CoAl-P (Figures 2B and S5B) contained vibrations typical for the CO_3_^2–^ band at 1349 cm^–1^ and the NO_3_^–^ band at 1396 cm^–1^.^38,43^

### Synthesis of CaAl LDH Modified with IL-P

**Figure 3:**
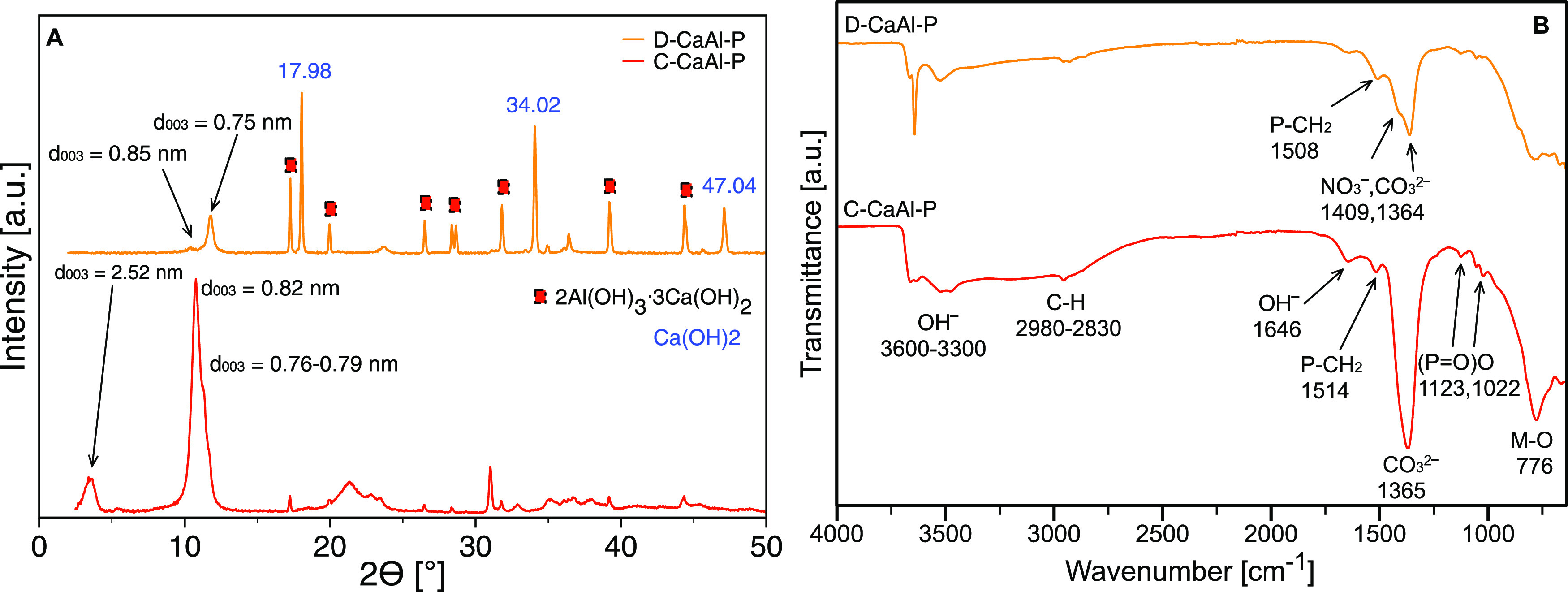
XRD pattern (A) and FTIR spectra (B) of CaAl LDH modified with IL-P (D—one-step direct coprecipitation, C—two-step coprecipitation/anion exchange, and U—two-step urea/anion exchange method).

The XRD pattern of D-CaAl-P showed basal spacings of 0.75 and 0.85 nm corresponding to CO_3_^2–^ and NO_3_^–^ anions, respectively (Figure 3A), which indicated unsuccessful intercalation of IL-P organic anions. Moreover, D-CaAl-P also contained calcium and aluminum hydroxide (Ca(OH)_2_, hydrogarnet 2Al(OH)_3_·3Ca(OH)_2_) byproducts. Due to a low LDH content in the product (ca. 37% via XRD), direct coprecipitation was found not to be a suitable method for the fabrication of IL-P-modified CaAl LDH.

Similarly, the urea method utilizing a typical 2/1 molar ratio of Ca/Al metal salts resulted in a white crystalline material of calcite (COD 9000095) and bayerite (COD 9010964) phases with no trace of a typical XRD pattern of CaAl LDH. Any attempt to optimize the synthesis (e.g., by increasing the pH value, using a large excess of urea, and prolonged heating) gave the aforementioned calcite/bayerite mixture. Despite the fact that the urea method has been successfully used for syntheses of a wide range of LDHs with different metals (CoAl LDH,^44^ ZnAl LDH,^45^ or MgAl LDH^46^) producing high-crystallinity materials with well-shaped particles, for the preparation of CaAl LDH, this method seems unusable.

Therefore, the two-step coprecipitation/anion exchange method was found to be the only successful way to prepare the IL-P-modified CaAl LDH. The XRD pattern of C-CaAl-P (Figure 3A) showed the presence of several LDH phases—with intercalated CO_3_^2–^ (*d*_003_ = 0.76–0.79) and NO_3_^–^ (*d*_003_ = 0.82 nm) and also the intercalated organic anion of IL-P (2θ of 3.5° corresponding to an interlayer spacing of 2.52 nm). The differences in interlayer distances between pristine (C-CaAl) and modified (C-CaAl-P) LDHs (Δ*d*_003_ = 1.65 nm) were related to organic anion sizes (Figure S6A).

The FTIR spectra of D-CaAl-P and C-CaAl-P (Figures 3B and S6B) confirmed the presence of IL-P: specific for phosphorus compound weak bands such as P–CH_2_ at 1508 cm^–1^ for D-CaAl-P and at 1514 cm^–1^ for C-CaAl-P, which were significantly shifted compared to the LDHs containing other cations (Mg^2+^ or Co^2+^), and (P=O)O at 1128 and 1028 cm^–1^. The asymmetrical and symmetrical vibrations of the CH_3_ and CH_2_ groups exhibited very low intensity. The FTIR spectrum of C-CaAl-P showed a broad CO_3_^2–^ band at 1365 cm^–1^, which may indicate the presence of NO_3_^–^ anions. It correlated well with the XRD pattern (Figure 3A), showing a diffraction line (*d*_003_ = 0.82 nm) corresponding to the intercalated NO_3_^–^ anion.

### Comparison of Different Synthetic Pathways for Preparation of IL-P-Modified LDH

Three different synthetic pathways and the prepared LDH are compared in Table 2. It was found that in the cases of two-step syntheses (the coprecipitation/anion exchange and urea/anion exchange methods), the course of calcination strongly depends on the type of LDH being prepared. During calcination, the LDH interlayered structure is distorted into amorphous mixed oxides (known as calcinated LDH).^47^ Then, when the calcinated LDH is dispersed in a suitable solution containing organic (large) anions, the LDH structure can be regenerated due to the presence of the memory effect, giving a parent hydroxide layer with intercalated organic anions.^39^ Herein, it was found that calcination was only possible for the modification of MgAl LDH, while in the cases of CoAl and CaAl LDHs, degradation occurred. Furthermore, the urea method was unsuccessful for the synthesis of pristine U-CaAl LDH, since the LDH structure was not formed.

**Table 2:** Comparison of Different Synthetic Pathways for Preparation of IL-P-Modified LDH

sample ID of LDH modified by IL-P	calcination	diffraction line (003) [nm]	intercalation of IL anion
One-Step Direct Coprecipitation Synthesis			
D-MgAl-P	not relevant	0.77, 3.84^a^	yes
D-CoAl-P	not relevant	0.77	no
D-CaAl-P	not relevant	0.75, 0.85^b^	no
Two-Step Coprecipitation/Anion Exchange Synthesis			
C-MgAl-P	necessary	0.76	no
C-CoAl-P	adverse	0.77	no
C-CaAl-P	adverse	0.76–0.79, 0.82^b^, 2.52^a^	yes
Two-Step Urea/Anion Exchange Synthesis^c^			
U-MgAl-P	necessary	0.75, 7.36^a^	yes
U-CoAl-P	adverse	0.75	no

a The (003) diffraction lines for intercalated and not intercalated LDHs are presented.

b The (003) diffraction lines for intercalated CO_3_^2–^ and NO_3_^–^ anions.

c The synthesis of U-CaAl LDH was not successful.

In all prepared LDHs, the intercalation of IL-P anions was negatively influenced by the formation of interlayered CO_3_^2–^ anions originating from atmospheric CO_2_ and from the decomposition of urea (in the case of the urea method; Table 2). Although an inert atmosphere and CO_3_^2–^-free starting salts were always used for LDH syntheses, it was not possible to completely prevent the presence of CO_3_^2–^ anions in the final LDHs, probably due to their very high affinity toward positively charged LDH layers (CO_3_^2–^ ≫ OH^–^ > Cl^–^ > NO_3_^–^).^36,39^

SEM images (Figures S7–S9) show the particle sizes of the produced LDHs in the order of C-MAl-P < D-MAl-P < U-MAl-P (M = Mg, Co, Ca). IL-P had the tendency to be adsorbed on the surface of the particles, resulting in the formation of agglomerates and preventing good dispersibility. To avoid this, intensive multistep washing had to be used.

The general effectiveness of LDH modification by IL-P was assessed based on the XRD results, where the shift of the (003) diffraction line reflects the intercalation of the organic fraction^48,49^ (Table 2). The intercalation of the IL-P anion was successfully achieved in the cases of D-MgAl-P, U-MgAl-P, and C-CaAl-P. Among them, C-CaAl-P exhibited uniform lateral sizes and a regular hexagonal shape of LDH sheets, whose morphology was only slightly changed after the modification step with IL-P (see TEM images in Figure S10). Therefore, C-CaAl-P was selected for further optimization and testing of its catalytic ability for εCL polymerization.

### Optimization of the Synthesis of IL-P-Modified CaAl LDH

**Figure 4:**
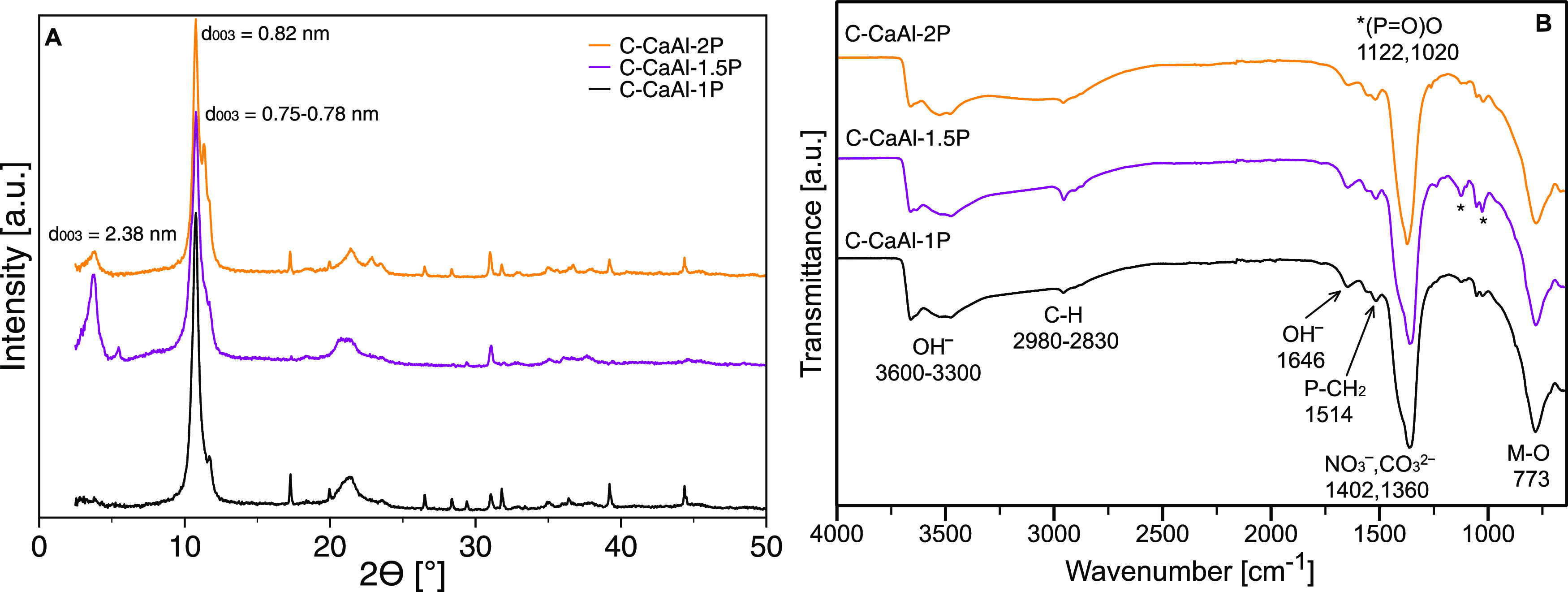
XRD pattern (A) and FTIR spectra (B) of CaAl LDH modified with IL-P prepared by the two-step coprecipitation method using 0% (C-CaAl-1P), 50% (C-CaAl-1.5P), and 100% (C-CaAl-2P) excess of IL-P regarding the AEC of LDH.

The two-step coprecipitation method was found to be the most suitable synthetic method for preparing a highly pure CaAl LDH with intercalated IL-P anions (C-CaAl-P). However, due to low efficiency, IL-P amounts had to be optimized using 0% (C-CaAl-1P), 50% (C-CaAl-1.5P), and 100% (C-CaAl-2P) excess of IL-P regarding the AEC of LDH. The XRD patterns (Figure 4A) revealed that 50% excess of IL-P (C-CaAl-1.5P) was found as the most efficient for the anion exchange as indicated by the presence of the most intensive peak at 2θ = 3.7° corresponding to the (003) reflection of the IL-P-intercalated LDH. The XRD patterns also showed that *d*_003_ = 0.82 and 0.75–78 nm correspond to phases containing the intercalated NO_3_^–^ and CO_3_^2–^ anions, respectively. The FTIR spectra (Figure 4B) showed the vibrations typical for LDH and IL-P. The different amounts of IL-P did not cause significant changes in the FTIR intensities of vibration bands.

To determine the thermal stability and content of organic anions for the optimized C-CaAl-P samples, TGA/DTA measurements were performed. First, C-CaAl-P and C-CaAl were measured, showing decomposition in three steps (Figure S11). Water removal proceeded up to ca. 200 °C, and the intercalated inorganic anions were released in the temperature range of 200–400 °C, above which dehydroxylation occurred. The additional decomposition step (with a maximum degradation rate at 443.4 °C) of the modified nanoparticles appeared for the C-CaAl-P sample at a temperature higher than the usual decomposition temperature of IL-P (ca. 380 °C, dashed line). The occurrence of the abovementioned step at this specific temperature was related to the presence of organic species in the modified LDH. The organic functionalization of Ca^2+^/Al^3+^ LDH resulted in a decrease of the nanoparticle’s thermal stability represented as the temperature at a 10% weight loss of the sample—the higher the temperature, the better the thermal stability, herein ca. 300 °C for C-CaAl and ca. 150 °C for C-CaAl-P.

**Figure 5:**
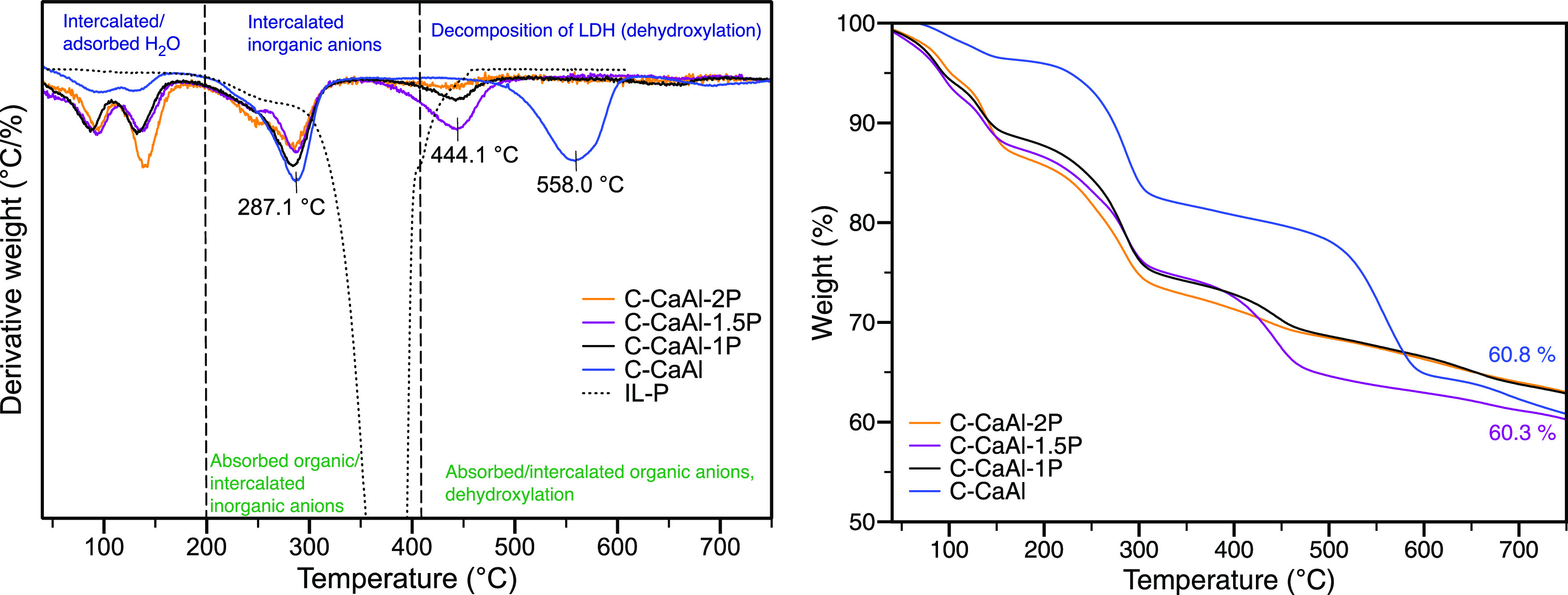
DTG (left) and TGA (right) of CaAl LDH modified with IL-P in a different ratio by the coprecipitation method.

According to the DTG curve of C-CaAl-P, one decomposition step at 444.1 °C (Figure 5) might be related to a release of the majority of IL-P, which was adsorbed on the LDH surface.^29^

### Determination of Water Content in LDH Modified with IL-P

The adsorbed and intercalated water molecules in LDH are able to initiate ROP of εCL,^29^ and their too-high content in LDH can negatively affect the course of polymerization and substantially decrease the targeted molar mass of produced PCL. Therefore, the vacuum drying process (100 °C) of C-CaAl LDH was studied in detail via XRD and TGA measurements to determine and minimize the water content. Regarding the DTG curves, moisture removal proceeded in two steps—adsorbed water evaporated up to 125 °C, and intercalated water was released in the temperature range from 125 to 180 °C. Then, the water content was calculated for each drying period (Table 3). The amount of water decreased with increasing drying time to 2.4 wt % after 6 h, and the content of intercalated water reached 0.6 wt % after 7 h and remained at the same level after 25 h. Concerning the adsorbed water content after 6, 7, and 25 h of drying, C-CaAl LDH was moisture-sensitive, inducing an increase in water amount after 7 and 25 h in comparison to the water amount after 6 h.

**Table 3:** Influence of the Vacuum Drying Time on C-CaAl LDH Water Content and C-CaAl LDH Crystalline Structure

drying time [h]	adsorbed water content [%]	intercalated water content [%]	total water content [%]	2θ angle [deg]
2	6.1	1.3	7.4	11.07; 12.05
4	1.7	0.9	2.6	12.11
6	1.7	0.7	2.4	12.11
7	2.1	0.6	2.7	12.15
25	1.9	0.6	2.5	12.16

The influence of drying time and the content of intercalated water on the XRD (003) plane position (basal spacing) was also studied (Table 3). Water removal from interlayer spacing induced narrowing LDH sheets, which corresponded to (003) plane shifts to higher angle values. The XRD measurement, more precisely the (003) plane position, can thus serve as a preliminary indicator of the amount of intercalated water in LDH. With correspondence to XRD and TGA results, the optimal drying time of LDH was determined as 7 h at 100 °C under vacuum before polymerization.

### Microwave-Initiated Ring-Opening Polymerization of ε-Caprolactone

**Figure 6:**
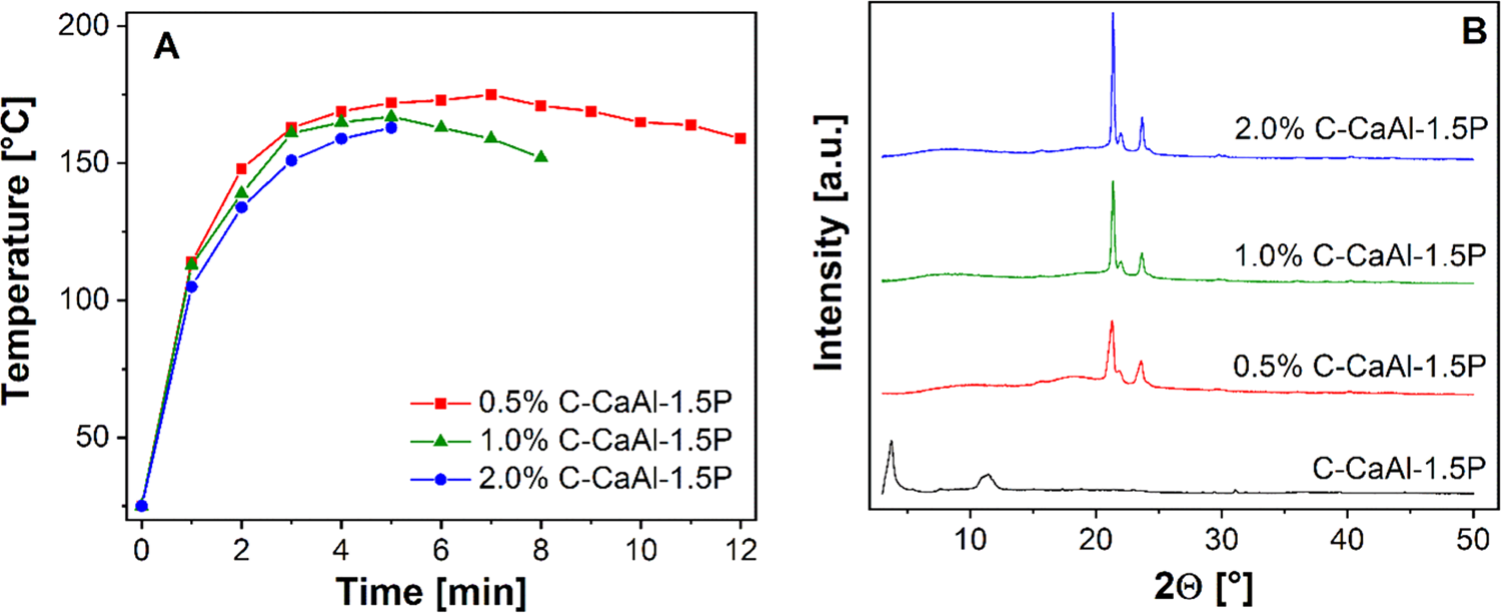
(A) Temperature profiles of the reaction mixtures of ε-caprolactone with different amounts of CaAl LDH modified with IL-P (C-CaAl-1.5P) under microwave irradiation using a constant power of 30 W. (B) XRD patterns of polycaprolactone containing different amounts of CaAl LDH modified with IL-P and prepared under microwave irradiation. Conditions of polymerization: constant power is 30 W and times are 5, 8, and 12 min for C-CaAl-1.5P contents of 0.5, 1, and 2 wt %, respectively.

The optimized C-CaAl-1.5P sample was selected for polymerization experiments to demonstrate the catalytic ability of IL-P immobilized on LDH nanoparticles. Microwave-assisted ROP of εCL in the presence of different amounts (0.5, 1, and 2 wt %) of C-CaAl-1.5P was performed using a constant microwave power of 30 W. Figure 6A shows fast heating of the reaction mixture, reaching the maximal temperature of ca 160–175 °C within 5–7 min. This demonstrated the high ability of the reaction mixture to absorb microwaves efficiently, mainly due to the presence of εCL.^9^ However, IL-P and water molecules, intercalated in the LDH structure, were also activated by microwave irradiation, which caused their rotation and release from the LDH galleries. The released water initiated the ROP of εCL, while IL-P anions catalyzed the progress of εCL polymerization.^29^ The high catalytic effect of the released IL-P anions led to the formation of a polymer (PCL) within a few minutes. The molar mass of the synthesized PCL decreased with the increasing content of C-CaAl-1.5P (Table 4), which was related to the increasing content of the released water acting as an initiator, as described previously.^29^ However, thanks to vacuum drying before polymerization (7 h at 100 °C), the total water content in LDH was significantly reduced. As a consequence, the average molar weight of the synthesized PCL (in the range of approximately 20–50 kg/mol, Table 4) was 1 order of magnitude higher than the previously published results using the non-dried LDH producing PCL with a molar mass range of 1.8–2.7 kg/mol.^9^ PCL with such a high molar mass already enables processing into the desired product (e.g., films), and therefore, the applied vacuum treatment prior to polymerization seems to be sufficient.

**Table 4:** Polymer Yield, Number Average Molar Mass (*M*_n_), and Dispersity (*M*_w_/*M*_n_) of Polycaprolactones Prepared by Microwave-Assisted ROP of ε-Caprolactone with Different Amounts of LDH Modified with IL-P (C-CaAl-1.5P) Using a Constant Power of 30 W

time [min]	C-CaAl-1.5P content [wt %]	polymer yield [wt %]	*M*_n_ [g/mol]	*M*_w_/*M*_n_
5	0.5	97	49 600	1.43
8	1.0	97	34 500	1.50
12	2.0	98	20 400	1.30

In addition to the catalytic effect of IL-P anions, their presence also affected the final structure of the PCL materials. The XRD patterns of all prepared PCL materials (Figure 6B) showed the disappearance of reflections typical for the basal spacing of the LDH, which indicated complete delamination and exfoliation of LDH particles. These results were in accordance with our previous study,^9^ showing that the exfoliated morphology of the PCL/LDH nanocomposites can only be achieved assuming the presence of intercalated IL anions in LDH galleries.

## Conclusions

In this work, the effects of LDH synthesis and structure on the immobilization of a phosphonium ionic liquid (IL) were studied. Three types of syntheses (one-step direct coprecipitation, two-step coprecipitation/ionic exchange, and two-step urea/ionic exchange) were applied for the preparation of M^2+^/Al^3+^ LDHs with three different M^2+^ cations (Mg, Co, Ca).

It was found that the successful preparation of M^2+^/Al^3+^ LDH particles with an intercalated organic phosphinate anion from a phosphonium ionic liquid (IL-P) depends on both the method used for LDH synthesis and the type of divalent metal cation (Co^2+^, Mg^2+^, or Ca^2+^) contained in the LDH structure. In the case of Co^2+^/Al^3+^ LDH, none of the methods succeeded in preparing IL-P anion-intercalated LDH. In contrast, Mg^2+^/Al^3+^ LDH with the intercalated IL-P anion can be prepared by direct coprecipitation or by the two-step urea/ionic exchange method. The IL-P anion-intercalated Ca^2+^/Al^3+^ LDH can only be synthesized by the two-step coprecipitation/anion exchange method.

Thus, LDH with intercalated IL-P anions can be prepared by all three synthesis routes. However, the appropriate method has to be selected with regard to the composition of the M^2+^ cation of LDH. The calcination step facilitating anion exchange in the two-step syntheses can only be applied for the preparation of Mg^2+^/Al^3+^ LDH, while it leads to undesirable degradation for Ca^2+^/Al^3+^ LDH and Co^2+^/Al^3+^ LDH.

The greatest obstacle preventing the achievement of a high degree of IL-P anion intercalation seems to be a high affinity of CO_3_^2–^ anions toward positively charged LDH sheets. The consequence of this is a mixed composition of intercalated anions, which is manifested by several basal spacings in XRD diffraction.

The organically modified Ca^2+^/Al^3+^ LDH exhibited a homogeneous morphology of LDH sheets and a high content of intercalated IL-P anions. Therefore, it was selected as the most promising catalyst for the microwave-accelerated ROP polymerization of ε-caprolactone. Prior to polymerization, vacuum drying had to be applied, reducing the interlayer water content. The dried LDH was then successfully tested as an efficient catalyst, leading to polymer formation within a few minutes. Moreover, thanks to the efficient intercalation of IL anions, homogeneous dispersion of fully exfoliated LDH nanoparticles within the polymer matrix was reached.

The abovementioned results thus emphasize the importance of successful intercalation of ionic species into the 2D-layered nanoparticles, which is a key presumption for successful polymerization and in situ preparation of polymer nanocomposites.

## Supporting Information Available

XRD pattern; FTIR spectra; SAXS data; SEM images; TEM images; DTG; and TGA (PDF)
