# Supplementary material for: Intercalation of Ionic Liquids into LDH Structures for Microwave-Accelerated Polymerizations
Source: Inorg Chem. 2023 Aug 28;62(36):14694–703. doi: 10.1021/acs.inorgchem.3c02021 (PMC10498490; doi:10.1021/acs.inorgchem.3c02021)
Supplement: Supplementary file 1 — ic3c02021_si_001.pdf [file ic3c02021_si_001.pdf]

Intercalation of ionic liquid into LDH structure for microwave-accelerated polymerizations

Hynek Beneš[1], Magdalena Konefał[1], Sonia Bujok[1], Ondřej Mrózek[2], Ewa Pavlova[1], Darina Smržová[2], Petra Ecorchard[2]*

[1]*Institute of Macromolecular Chemistry, Czech Academy of Sciences, Heyrovského nám. 2, Prague 6, 162 00, Czech Republic*

[2]*Institute of Inorganic Chemistry of the Czech Academy of Sciences, Husinec-Řež 1001, 250 68 Řež, Czech Republic*

Corresponding author: ecorchard@iic.cas.cz

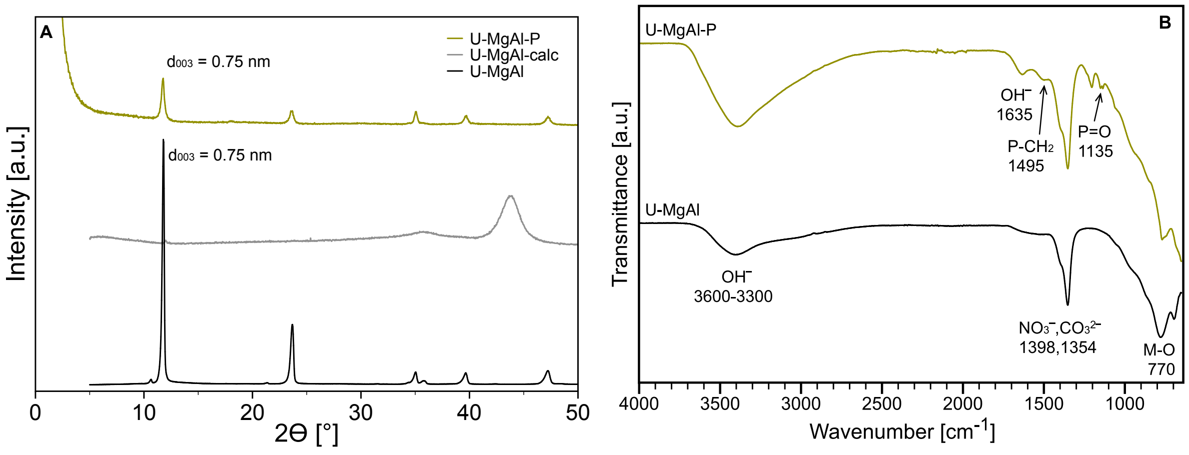

Fig. S1 XRD pattern (A) and FTIR spectra (B) of MgAl LDH modified with IL-P by the two-step urea / anion exchange method

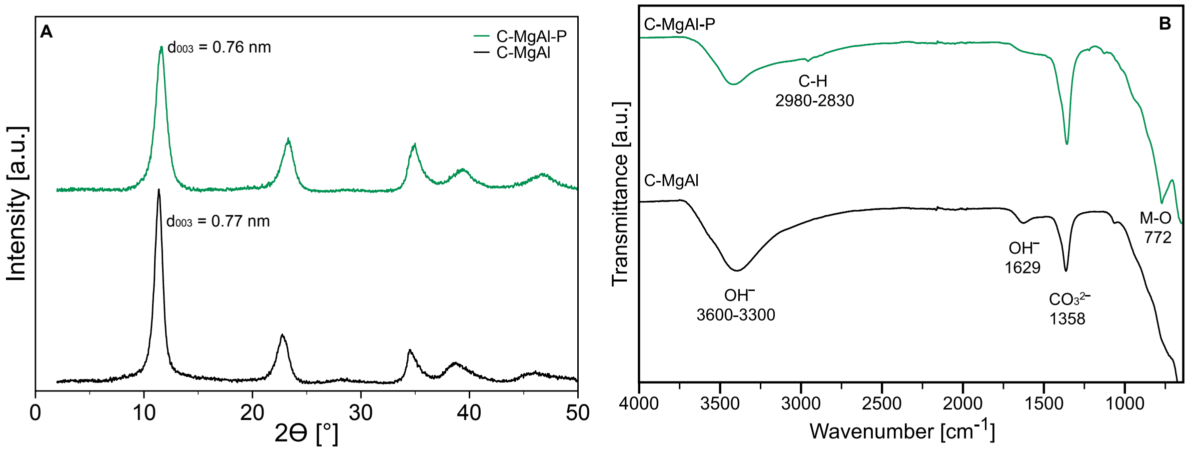

Fig. S2 XRD pattern (A) and FTIR spectra (B) of MgAl LDH modified with IL-P by the two-step co-precipitation / anion-exchange

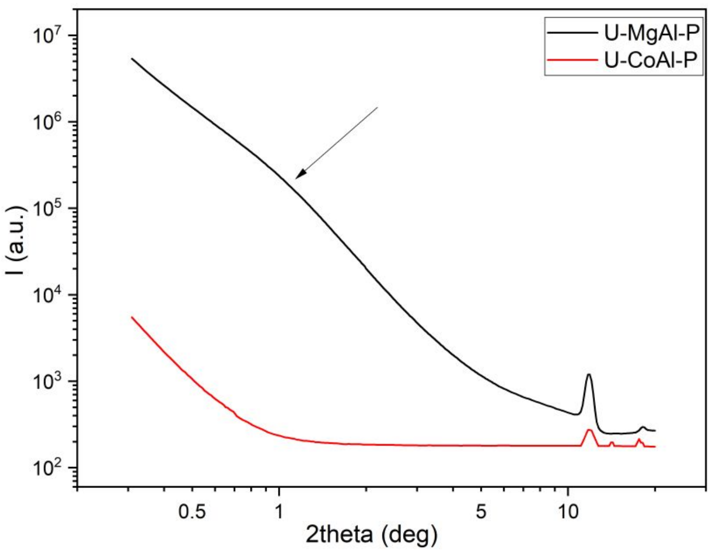

Fig. S3 SAXS data of MgAl and CoAl LDH modified with IL-P by the two-step urea / anion exchange method. The results showed the low intensity of very broad reflection for U-MgAl-P at approx. 1.2° 2θ (7.36 nm) and no diffraction line for U-CoAl-P corresponding to intercalation of IL-P.

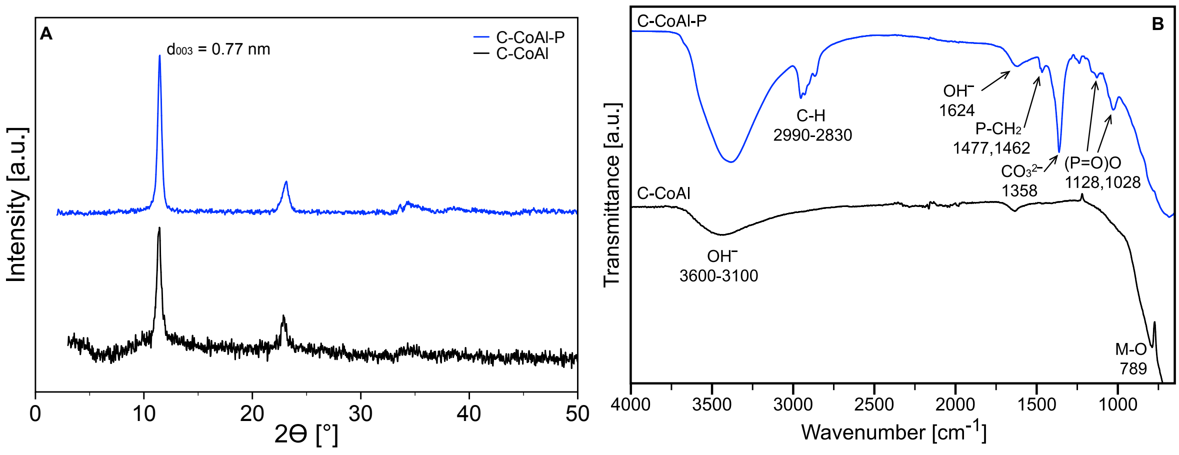

Fig. S4 XRD pattern (A) and FTIR spectra (B) of CoAl LDH modified with IL-P by the two-step co-precipitation / anion-exchange

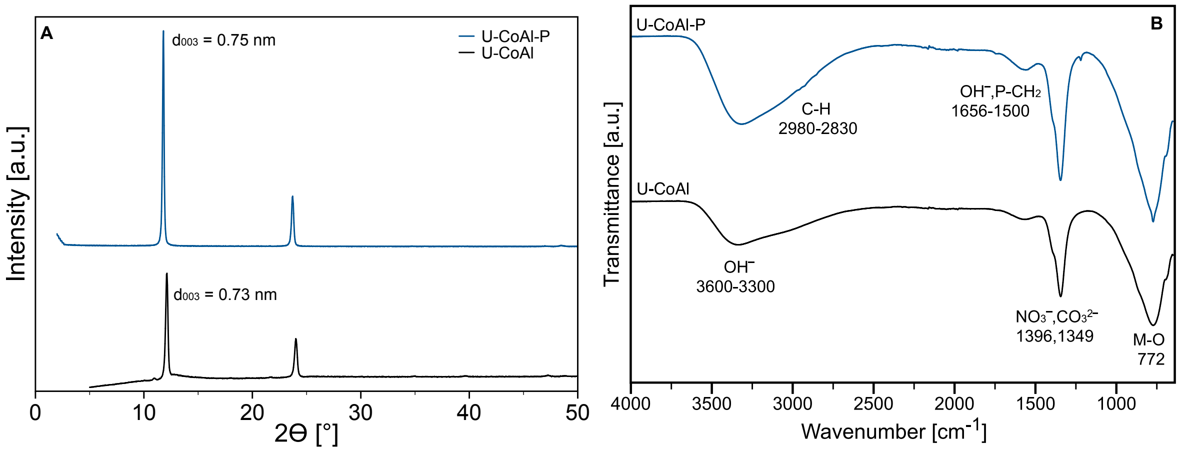

Fig. S5 XRD pattern (A) and FTIR spectra (B) of CoAl LDH modified with IL-P by the two-step urea / anion exchange method

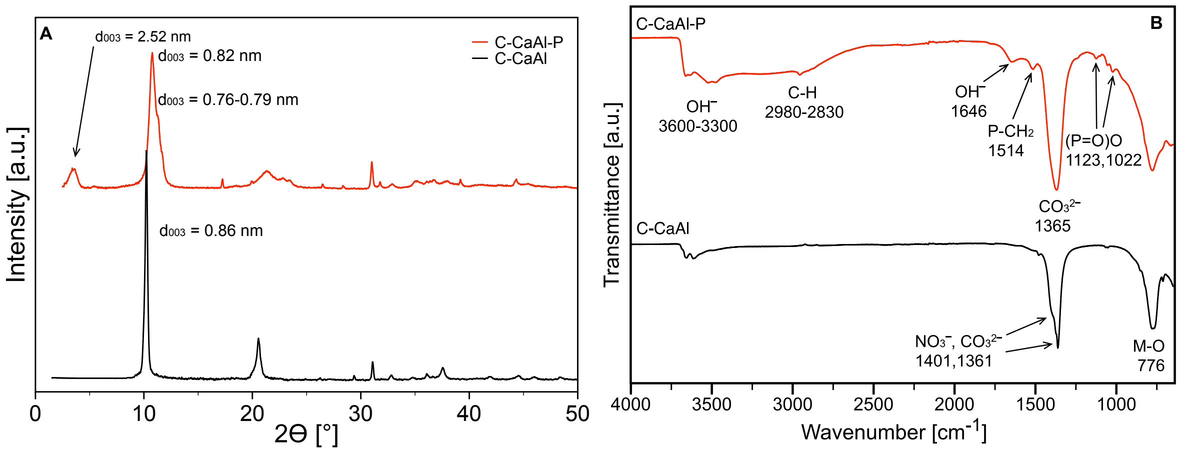

Fig. S6 XRD pattern (A) and FTIR spectra (B) of CaAl LDH modified with IL-P by the two-step co-precipitation / anion-exchange

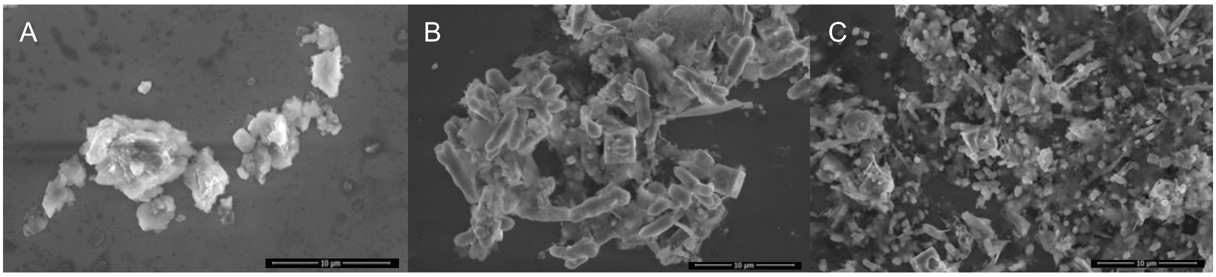

Fig. S7 SEM images (A) D-MgAl-P, (B) D-CoAl-P, and (C) D-CaAl-P, samples modified with IL-P by direct synthesis

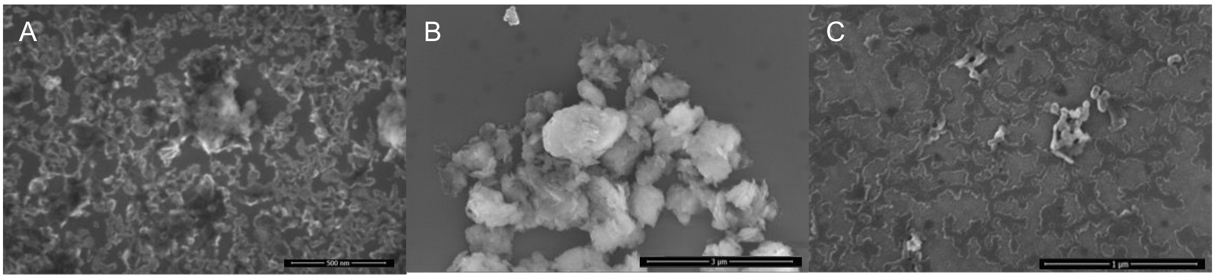

Fig. S8 SEM images (A) C-MgAl-P, (B) C-CoAl-P, and (C) C-CaAl-P, samples modified with IL-P by co-precipitation method

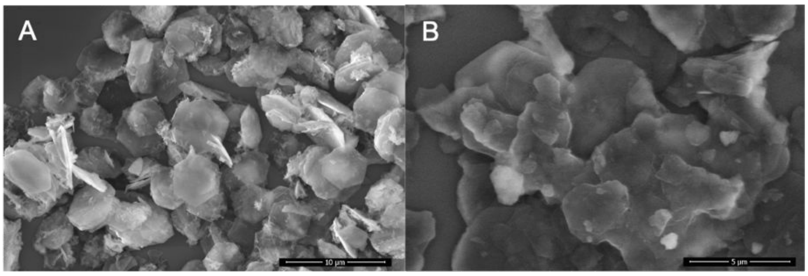

Fig. S9 SEM images (A) U-MgAl-P and (B) U-CoAl-P samples modified with IL-P by urea method

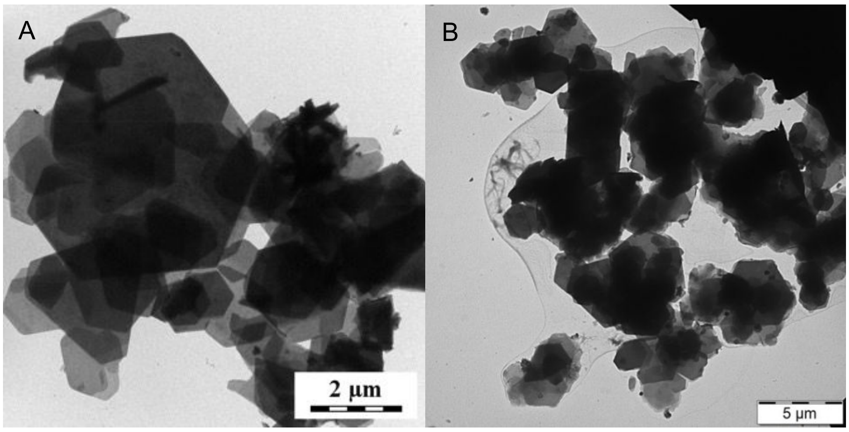

Fig. S10 TEM images (A) C-CaAl and (B) C-CaAl-P

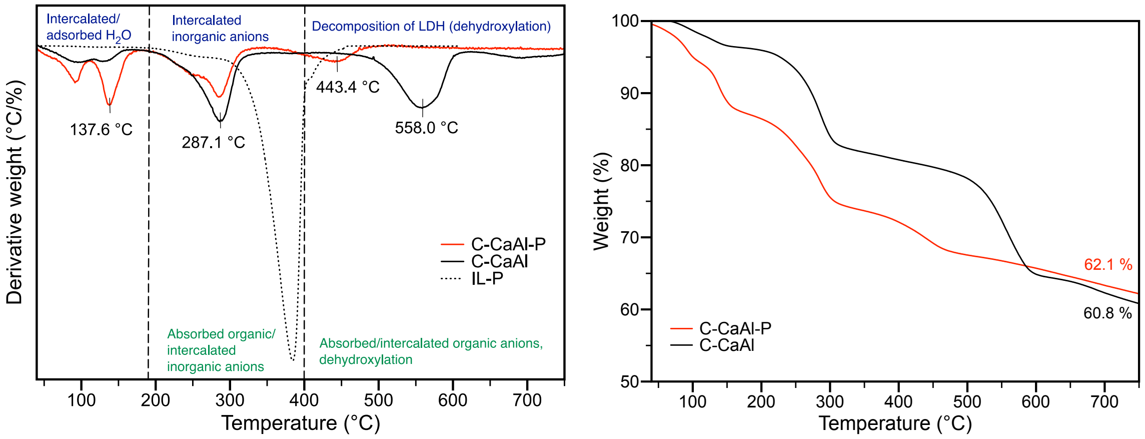

Fig. S11. DTG (left) and TGA (right) of CaAl LDH modified with IL-P by the two-step co-precipitation / anion-exchange
